# A Lentiviral Vector Expressing Japanese Encephalitis Virus-like Particles Elicits Broad Neutralizing Antibody Response in Pigs

**DOI:** 10.1371/journal.pntd.0004081

**Published:** 2015-10-05

**Authors:** Mélissanne de Wispelaere, Meret Ricklin, Philippe Souque, Marie-Pascale Frenkiel, Sylvie Paulous, Obdulio Garcìa-Nicolàs, Artur Summerfield, Pierre Charneau, Philippe Desprès

**Affiliations:** 1 Interactions Moléculaires Flavivirus-Hôtes, Institut Pasteur, Paris, France; 2 Institute of Virology and Immunology, Mittelhäusern, Switzerland; 3 Virologie Moléculaire et Vaccinologie, Institut Pasteur, Paris, France; 4 Department of Infectious Diseases and Pathobiology, Vetsuisse Faculty, University of Bern, Bern, Switzerland; George Mason University, UNITED STATES

## Abstract

**Background:**

Japanese encephalitis virus (JEV) is the major cause of viral encephalitis in Southeast Asia. Vaccination of domestic pigs has been suggested as a “one health” strategy to reduce viral disease transmission to humans. The efficiency of two lentiviral TRIP/JEV vectors expressing the JEV envelope prM and E glycoproteins at eliciting protective humoral response was assessed in a mouse model and piglets.

**Methodology/Principal Findings:**

A gene encoding the envelope proteins prM and E from a genotype 3 JEV strain was inserted into a lentiviral TRIP vector. Two lentiviral vectors TRIP/JEV were generated, each expressing the prM signal peptide followed by the prM protein and the E glycoprotein, the latter being expressed either in its native form or lacking its two C-terminal transmembrane domains. *In vitro* transduction of cells with the TRIP/JEV vector expressing the native prM and E resulted in the efficient secretion of virus-like particles of Japanese encephalitis virus. Immunization of BALB/c mice with TRIP/JEV vectors resulted in the production of IgGs against Japanese encephalitis virus, and the injection of a second dose one month after the prime injection greatly boosted antibody titers. The TRIP/JEV vectors elicited neutralizing antibodies against JEV strains belonging to genotypes 1, 3, and 5. Immunization of piglets with two doses of the lentiviral vector expressing JEV virus-like particles led to high titers of anti-JEV antibodies, that had efficient neutralizing activity regardless of the JEV genotype tested.

**Conclusions/Significance:**

Immunization of pigs with the lentiviral vector expressing JEV virus-like particles is particularly efficient to prime antigen-specific humoral immunity and trigger neutralizing antibody responses against JEV genotypes 1, 3, and 5. The titers of neutralizing antibodies elicited by the TRIP/JEV vector are sufficient to confer protection in domestic pigs against different genotypes of JEV and this could be of a great utility in endemic regions where more than one genotype is circulating.

## Author Summary

Japanese encephalitis virus is the etiologic agent of the most medically important viral encephalitis in South Asia with thousands of deaths per year. The virus is maintained in an enzootic cycle between *Culex* mosquitoes and amplifying vertebrate hosts, such as wild boars and pigs. Vaccination of domestic pigs has been suggested as a strategy to reduce viral disease transmission to humans, in line with the now-called “One Health” concept. Lentiviral gene transfer vectors represent a novel vaccination platform with an unprecedented ability to induce robust humoral immunity in various animal species. In our study, we demonstrated that immunization of pigs with a recombinant lentiviral vector expressing virus-like particles of Japanese encephalitis virus is particularly efficient at eliciting specific humoral immunity. The titers of neutralizing antibodies elicited by the lentiviral vector are sufficient to confer protection in domestic pigs against the different genotypes of Japanese encephalitis virus observed in Asia.

## Introduction

Mosquito-borne Japanese encephalitis virus is a member of the *Flavivirus* genus in the *Flaviviridae* family [1–4]. Flaviviruses contain a positive single-stranded RNA genome encoding a polyprotein that is processed into three structural proteins, the capsid (C), the precursor of membrane (prM) and the envelope (E), and seven non-structural proteins NS1 to NS5 [4]. Viral assembly occurs in the lumen of the endoplasmic reticulum: the nucleocapsids associate with prM-E heterodimers to form an immature JEV virion. The latter transits through the secretory pathway, where it is matured through cleavage of prM into the membrane (M) protein by furin in the *trans*-Golgi [4]. Additionally, JEV produces virus-like particles (VLPs), which are assembled solely from prM and E proteins, and undergo the same maturation process as genuine viral particles [5]. These VLPs can be produced in the absence of any other viral component [5].

JEV is the etiologic agent of the most important viral encephalitis of medical interest in South Asia, with an incidence of 50,000 cases and about 10,000 deaths per year [1, 3, 6]. About 20 to 30% of the symptomatic human cases are fatal, while 30 to 50% of survivors can develop long-term neurologic *sequelae*. JEV is usually maintained in an enzootic cycle between *Culex tritaeniorhynchus* mosquitoes and amplifying vertebrate hosts, such as waterbirds and domestic pigs [1, 3, 7]. Horses and humans are thought to be dead-end hosts, since they do not develop a level of viremia sufficient to infect mosquitoes [7]. In the past decades, there has been an expansion of the geographic distribution of JEV in Asia and a possible introduction of JEV into Europe has been documented recently [6, 8]. Phylogenetic studies based on the viral envelope protein sequences allow the division of JEV strains into genotypes G1 to G5 [1, 3, 9–15]. Initially, most of the circulating strains of JEV belonged to G3 and were at the origin of major epidemics in Southeast Asian countries. Recently a shift in prevalence from JEV G3 to G1 has been observed in several Asian countries, while some strains of JEV G5 have been occasionally isolated in China and South Korea [9–16].

We previously demonstrated that both integrative and non-integrative lentiviral vectors are promising vaccination vectors against arboviruses such as West Nile virus (WNV), a neurotropic Flavivirus that belongs to the JEV serocomplex [17, 18]. Immunization with a single minute dose of recombinant lentiviral TRIP vectors that express the soluble form of WNV E protein resulted to a robust protection against a lethal challenge with WNV in mice [17, 18]. The currently used lentiviral delivery vectors, mostly derived from human deficiency virus-1 (HIV-1), allow *in vivo* stable transduction of dendritic cells. This allows a sustained antigen presentation through the endogenous pathway, which in turn elicits robust both humoral and cellular adaptative immunity [19–21].

Humoral immunity plays a pivotal role in protection against JEV infection [22–25] and consequently, the elicitation of a protective antibody response is critical in the development of safe JEV vaccines [25]. In the present study, we evaluated the ability of two lentiviral vectors TRIP/JEV expressing JEV G3 prM and E to induce a protective humoral immune response against JEV infection in a mouse model and in pigs. Both TRIP/JEV.prME vectors were efficient at producing broad anti-JEV neutralizing antibodies in a mouse model. Immunization of piglets with a TRIP/JE vector expressing JEV VLPs elicited high titers of specific neutralizing antibodies that could give a sufficient protection against different JEV genotypes.

## Methods

### Cells and antibodies

Mosquito *Aedes albopictus* C6/36 cells were maintained at 28°C in Leibovitz medium (L15) supplemented with 10% heat-inactivated fetal bovine serum (FBS). Vero cells and were maintained at 37°C in Dulbecco’s modified Eagle medium (DMEM) supplemented with 5% FBS. BHK-21, SK-N-SH, and HEK-293T cells were maintained at 37°C in DMEM supplemented with 10% FBS. Highly purified mouse anti-*pan* Flavivirus E monoclonal antibody (mAb) 4G2 was produced by RD Biotech (Besançon, France). Mouse mAb anti-JEV NS5 was kindly provided by Y. Matsuura [26]. Antibodies against Calnexin and SNAP-tag were purchased from Enzo Life Sciences and New England Biolabs, respectively. Horseradish peroxidase (HRP)-conjugated goat anti-mouse IgG and anti-rabbit IgG antibodies were obtained from Bio-Rad Laboratories. HRP-conjugated goat anti-pig antibody was obtained from Bethyl Laboratories. Alexa Fluor 488-conjugated goat anti-mouse IgG antibody was obtained from Jackson ImmunoResearch.

### JEV strains

The JEV G1 strain G1 CNS769_Laos_2009 [27] was kindly provided by R. Charrel [27]. The JEV G3 strain Nakayama was obtained from the National Collection of Pathogenic Viruses (NCPV, Salisbury, UK) and passaged twice on Vero cells. The molecular clone of JEV G3 strain RP-9 was kindly provided by Y-L. Lin [28] and modified to produce pBR322(CMV)-JEV-RP9, as described previously [29]. To produce infectious virus, the molecular clone was transfected into HEK-293T cells using Lipofectamine 2000 (Life Technologies). At three days post-transfection, viral supernatants were collected and used to infect C6/36 cells in order to grow final virus stocks for experiments. The chimera JEV S-g5/NS-g3 (G5/G3) which express the structural proteins C, prM, and E from the JEV G5 strain XZ0934 fused to the nonstructural proteins of JEV G3 RP-9 has been already described [29]. The chimeric JEV S-g1/NS-g3 (G1/G3) virus containing the structural protein region from the JEV G1 CNS769_Laos_2009 fused to the RP-9 nonstructural protein region was produced as follows. A silent mutation that created a unique restriction site (*Afl*II) at position 2208–2213 (residues 705 and 706 of the viral polyprotein) was introduced directly in pBR322(CMV)-JEV-RP9 through PCR mutagenesis using primers 5’-actgggaaaggctttcacgaccactcttaagggtgctcagagac-3’ and 5’-gtctctgagcacccttaagagtggtcgtgaaagcctttcccagt-3’ (the *Afl* II site is underlined). The resulting pBR322(CMV)-JEV-RP9(*Afl* II) plasmid was used as template to generate the chimeric JEV. The fragment corresponding to nucleotides 114 to 2213 and flanked by the unique sites *Apa*I and *Afl*II was substituted with the homologous fragments of JEV G1 strain (nucleotides 115–2214). Both chimeric viruses were produced through transfection of the cDNA infectious clone, as described for JEV G3 strain RP-9.

### Generation of recombinant lentiviral vectors

For the construction of recombinant lentiviral vectors expressing JEV proteins, modifications that optimize the expression of prM and E genes in mammalian cells were done on the original sequence of JEV strain RP-9 of G3 using a synthetic gene (Genecust, Lux.). The mammalian codon-optimized sequence coding for prM signal peptide followed by prM and E glycoproteins was cloned into the *Bam*H I and *Xho* I restriction sites of the pTRIPΔU3CMV plasmid, to generate pTRIPΔU3CMV/JEV.prME. The optimized sequence was further modified by mutagenesis PCR to generate pTRIPΔU3CMV/JEV.prME^ΔTM^ that contains the genes encoding prM and E lacking its two transmembrane domains (E^ΔTM^).

Lentiviral particles were produced by transient calcium co-transfection of HEK-293T cells as described previously [30], but with the following modifications: at 24h hours post-transfection, the cell culture medium was replaced by serum-free DMEM. Supernatants were collected at 48 hours post-transfection, clarified by several rounds of low-speed centrifugation, and stored at -20°C. The recombinant lentiviral vectors were pseudotyped with VSV-G envelope protein of serotype Indiana (IND) or New Jersey (NJ) [31]. In the resulting vectors TRIP/JEV.prME and TRIP/JEV.prME^ΔTM^ the CMV immediate early promoter (CMVie) drives the constitutive expression of recombinant JEV proteins. The TRIP/JEV vector stocks were titrated by real-time PCR on cell lysates from transduced HEK-293T cells and expressed as transduction unit (TU) per ml [17,18]. Titers of non-concentrated TRIP/JEV.prME vector bearing IND or NJ VSV.G envelope protein were 6.8 log_10_ TU.mL^-1^ and 6.2 log_10_ TU.mL^-1^, respectively.

Titers of lentiviral TRIP/JEV.prME^ΔTM^ vector bearing IND or NJ VSV.G envelope protein were 7.1 log_10_ TU.mL^-1^ and 6.2 log_10_ TU.mL^-^1, respectively. Lentiviral vector stocks were adjusted by dilution in sterile PBS and were inoculated in mice or pigs without further concentration.

### Purification of JEV VLPs

Human HEK-293T cells were transduced with recombinant lentiviral vectors. The supernatants were collected at 2 days post-transduction and clarified by centrifugation at 3,000g for 5 min at 4°C. The clarified supernatant was loaded over a 15% sucrose cushion in TNE buffer (10 mM Tris-HCl [pH 7.5], 2.5 mM EDTA, 50 mM NaCl), and centrifuged at 100,000g for 2.5 h at 4°C. The supernatants were discarded, and the purified virus-like particles (VLPs) were suspended in 50 μl of TNE buffer.

### Focus-forming assay

For titration of JEV infectivity, focus-forming assays (FFA) were performed on BHK-21 cells as previously described [29]. Virus titers were given in focus forming units per ml (FFU.mL^-1^).

### Production of JEV antigens

Large flasks of Vero cell monolayers were inoculated with JEV at low multiplicity of infection. The supernatant fluids of JEV-infected (JEV antigen) or mock-infected (normal cell antigen or NCA) cells were harvested and clarified. The supernatants were precipitated with 7% ^w^/_v_ PEG 6,000 (Fluka), centrifuged, and the viral pellet was suspended in cold PBS supplemented with 0.1% ß-propiolactone in 0.1 M Sorensen buffer (pH 9.0) for JEV inactivation. The working dilution of inactivated JEV antigen (1:200) was estimated based on an « in-house » indirect ELISA using well-characterized human positive JEV serum samples and already validated JEV antigen.

The DES expression system (Life Technologies) was required for the production of recombinant viral antigens in *Drosophila* S2 cells. A synthetic gene coding for prM followed by E^ΔTM^ from JEV G3 strain SA-14 was cloned into the shuttle vector pMT/BiP/SNAP, a derived pMT/BiP/V5-HisA plasmid (Life Technologies) in which the SNAP-tag sequence (Covalys BioSciences AG) had been inserted in frame with the insect BiP signal peptide. The resulting plasmid pMT/BiP/JEV.prME^ΔTM^-SNAP encodes prM followed by E^ΔTM^ in fusion with the N-terminus of SNAP-tag. The recombinant domain III from the E protein (EDIII) of JEV G1 strain JaNAr0102/Japan/2002/Mosquito, JEV G3 strain GP05, and JEV G5 strain 10–1827 were fused in frame to the C-terminus of SNAP-tag into the plasmid pMT/BiP/SNAP. The resulting plasmids pMT/BiP/JEV.prME^ΔTM^-SNAP and pMT/BiP/SNAP-JEV.EDIII were transfected into S2 cells to establish stable cell lines S2/JEV.prME^ΔTM^-SNAP and S2/SNAP-JEV.EDIII for G1, G3, and G5 as previously described [29]. The production and the purification of recombinant viral antigens from stable S2 cell lines were performed as previously described [28].

### Immunodetection of viral proteins

Western blot assay was essentially performed as previously described [29]. For immunofluorescence (IF) assay, cells were fixed with 3.2% paraformaldehyde in PBS and permeabilized with 0.1% Triton X-100 in PBS. JEV E protein was detected with the mAb 4G2, followed by incubation with AlexaFluor488-conjugated secondary antibody. The cover slips were mounted with ProLong Gold Antifade Reagent with DAPI (Life Technologies). The slides were examined using a fluorescent microscope (Axioplan 2 Imaging, Zeiss).

### Mouse experiments

Groups of 6-week-old female BALB/c mice (*n* = 6) were intraperitoneally (i.p.) inoculated with recombinant lentiviral vectors in 0.1 ml DPBS supplemented with 0.2% endotoxin-free serum albumin. Animals were bled by puncturing at the retro-orbital sinus level. A very low individual variability exists within each group of mice inoculated with recombinant lentiviral vectors justifying the use of pooled sera in subsequent experiments [18]. A control group of 3-week-old female BALB/c mice (*n* = 6) was inoculated with 3 log_10_ FFU of JEV G3 (strain RP-9) and immune sera was collected and pooled at 21 days post-inoculation.

For passive seroprotection experiments, pooled immune sera were transferred i.p. into groups of 3-week-old C57BL/6 female mice (*n* = 6 or 12) one day before a challenge with 10 FFU of JEV G3 (strain RP-9) inoculated by the i.p. route. The challenged mice were monitored for signs of morbidity and mortality. Euthanasia was applied on animals showing the symptoms of viral encephalitis.

### Piglet experiments

Groups of 7-week-old specific pathogen free Swiss Land Race piglets from in-house breeding were housed in groups, and an adaptation time to the new environment of one week was given before starting the experiment.

For immunization, the TRIP/JEV.prME lentiviral vector was diluted to a final volume of 0.5 ml with PBS (Life Technologies). Immunization with the TRIP/GFP lentiviral vector was used as a negative control [18]. From a group of 5 piglets, four were vaccinated intramuscularly with various doses of the TRIP/JEV.prME vector and one was injected with the equivalent dose of control lentiviral vector TRIP/GFP. Immunized animals were bled before the first vaccination and then weekly until the end of the experiment. Four weeks after the first vaccination, all animals got a booster vaccination with the same dose of recombinant lentiviral vectors as at the first time point. For ethical reasons no lethal challenge was performed as protection in pigs. As a control, 3 animals were inoculated by the oronasal route with 7 log_10_TCID_50_ of live JEV Nakayama G3. All pigs developed temporary fever and viremia and recovered completely after 4–6 days. The animal sera were examined weekly for anti-JEV antibody.

### Indirect ELISA and neutralization test

Indirect ELISA measured the production of anti-JEV IgGs in immunized mice and piglets. A series of 96-well ELISA plates (Nunc) was coated with 0.1 ml of inactivated native JEV antigen or highly purified recombinant JEV antigens diluted in PBS at the concentration of 1 μg.mL^-1^ at 4°C overnight. NCA and SNAP served as negative control antigens. Indirect ELISA were performed as previously described (29). The Immune Status Ratio (ISR) of each group of immunized mice is obtained by dividing the average of JEV antigen OD_450_ values by the average control antigen OD_450_ values. The end-point titers of anti-JEV antibodies in mouse sera were calculated as the reciprocal of the last dilution of serum having ISR value > 3.0. Pig sera were tested as described for the mice, using HRP-conjugated goat anti-pig antibody as a secondary antibody. Pig sera obtained prior immunization were used as a negative control.

Neutralizing ability of mouse and pig serum antibodies against JEV was determined by focus (FRNT) or plaque (PRNT) reduction neutralization tests on Vero cells, respectively. Mouse serum samples from each group were pooled. Pig sera were tested individually in triplicates starting at a 1:5 serum dilution. Pooled mouse or individual pig serum were two-fold serial diluted in DMEM supplemented with 2% FBS, with a starting dilution of 1:10, and incubated for 2 h at 37°C with an equal volume of viral suspension containing 100 FFU of JEV. The end-point titer was calculated as the reciprocal of the highest serum dilution tested that reduced the number of FFU (FRNT_50_) or PFU (PRNT_50_) by 50%.

### Statistical analysis

A Log-rank (Mantel-Cox) test was used to compare survival data. Antibody levels between groups of immunized pigs were compared by Mann Whitney U test and the level of significance was set at 5%. GraphPad Prism (GrapPad Software Inc. La Jolla, CA, USA) was used for all statistical analysis.

### Ethics statement

All mice were housed under pathogen-free conditions at the Institut Pasteur animal facility. The protocols and subsequent experiments were ethically approved by the CETEA (Ethic Committee for Control of Experiments on Animals: http://cache.media.enseignementsup-recherche.gouv.fr/file/Encadrement_des_pratiques_de_recherche/58/1/Charte_nationale_portant_sur_l_ethique_de_l_experimentation_animale-version_anglaise_243581.pdf) at the Institut Pasteur (C2A N°89/CETEA) with the reference n°2013–0071 and declared to the French *Ministère de l’Enseignement Supérieur et de la Recherche* (reference n° 000762.1) in accordance with the *articles R*.*214-24 et R*.*214-125 du Code Rural et de la Pêche Maritime* and *R*.*214-120 du décret n°2013–118 du 1er février 2013* in France. Experiments were conducted following the guidelines of the Office Laboratory of Animal Care at the Institut Pasteur. The protocols and subsequent experiments on pigs were ethically approved by the cantonal ethical committee of Bern (number BE 118–13) and the FSVO (Federal Food Safety and Veterinary Office, website: http://www.blv.admin.ch/index.html?lang=en) in Switzerland. Pig experiments were conducted following the guidelines of Swiss Animal Welfare Regulations (Veterinary Service of LANAT).

### Accession numbers

The Genbank accession numbers of JEV strains SA-14, JaNAr0102/Japan/2002/Mosquito, GP05 and 10–187 are M55506, AY377577, FJ979830, and JN587258, respectively.

## Results

### TRIP/JEV vectors expressing JEV prM and E

**Fig 1:**
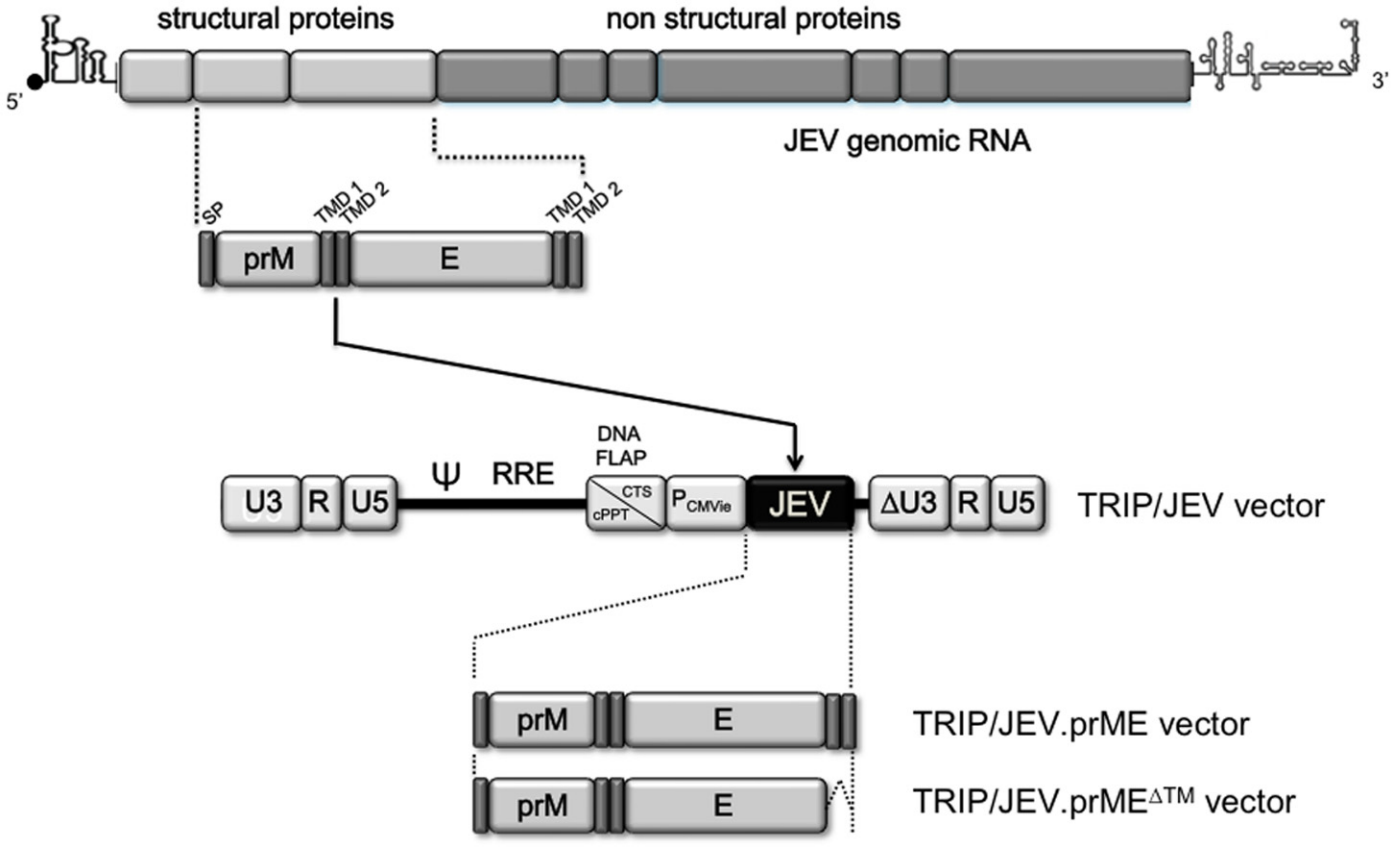
Schematic representation of recombinant lentiviral TRIP/JEV vectors. The organization of JEV genomic RNA is shown in top. The codon-optimized sequence encoding prM and E from JEV G3 strain RP-9 was cloned into a TRIP lentiviral vector under the control of human cytomegalovirus immediate early promoter (P_CMVie_). The TRIP/JEV.prME vector includes the signal peptide sequence of prM (SP) followed by the entire prM and E gene regions of JEV strain RP-9 of G3. The TRIP/prME^ΔTM^ vector includes the same JEV sequence except that E was deleted from its two transmembrane domains TMD1 and TMD2.

We have reported earlier that a single minute dose of a non-replicative lentiviral vector expressing the soluble form of WNV E glycoprotein induced a robust protective humoral response in a mouse model of WNV encephalitis [17, 18]. To assess the potential of lentiviral vectors expressing JEV proteins at eliciting protective humoral response against JEV infection, a mammalian codon-optimized gene encoding prM and E from the JEV strain RP9 of G3 was inserted into the lentivirus TRIP vector (Fig 1). We generated two lentiviral vectors, expressing the prM signal peptide followed by the prM protein and the E glycoprotein, the latter being expressed either in the native form (TRIP/JEV.prME) or lacking its two C-terminal transmembrane domains (TRIP/JEV.prME^ΔTM^). In these constructs, prM contributes to the folding, stability, and efficient secretion of the glycoprotein E. Lentiviral vectors which expressed JEV proteins were pseudotyped with VSV-G protein of the IND serotype. Non-replicative TRIP/JEV.prME and TRIP/JEV.prME^ΔTM^ particles were produced from HEK-293T cells, achieving titers of about 7 log_10_ TU.mL^-1^.

**Fig 2:**
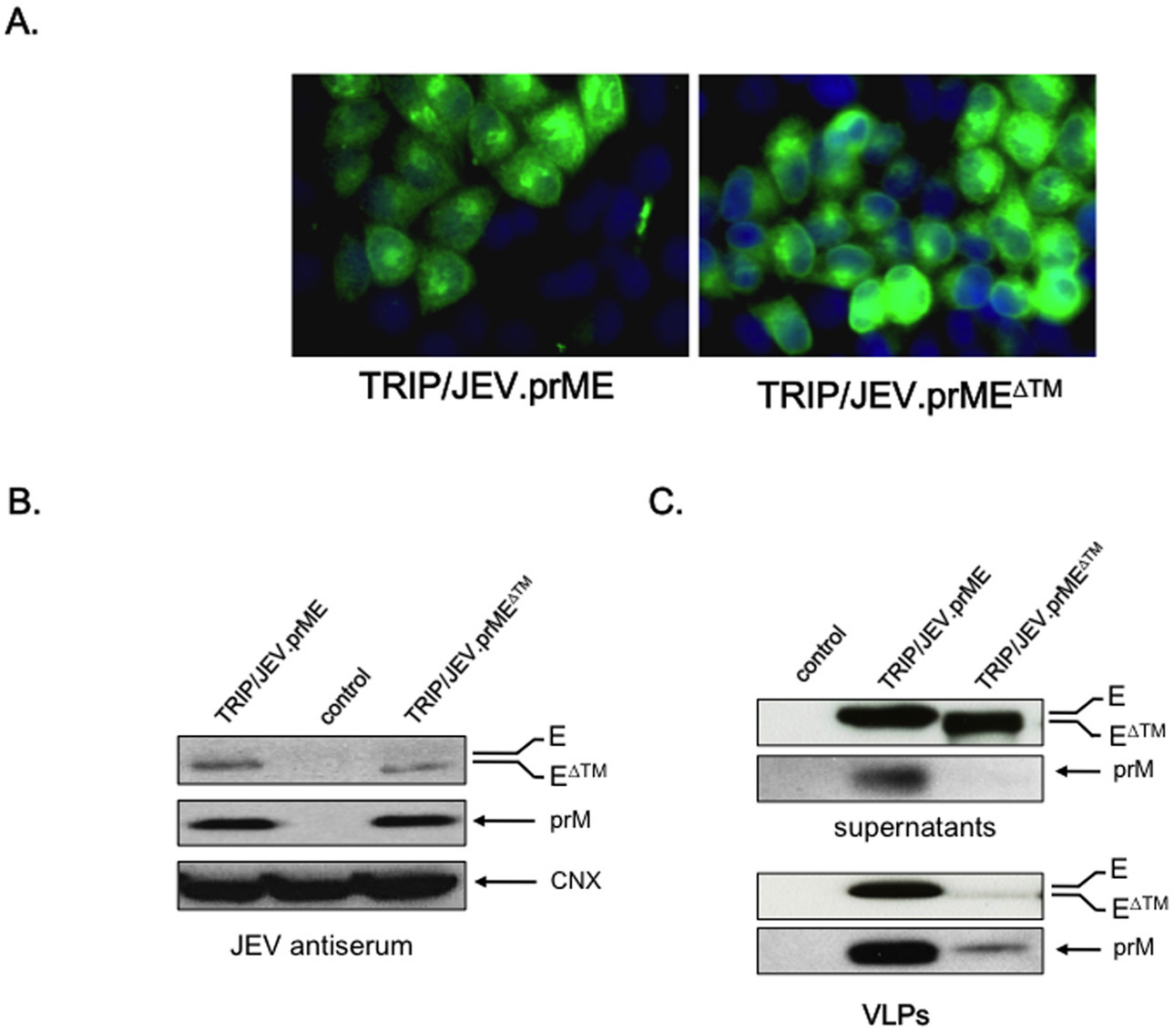
Transduction of HEK-293T cells with lentiviral TRIP/JEV vectors. *In* (A), immunofluorescence analysis of transduced cells using the anti-E mAb 4G2 as primary antibody. *In* (B, C), immunoblot analysis of prM and E accumulation in lysates (B) or supernatants (C) of cells transduced with TRIP/JEV vectors. TRIP/GFP vector served as a control. The intracellular prM and E were detected with a mouse polyclonal serum directed against JEV strain RP-9 (JEV antiserum). Detection of calnexin (CNX) served as a loading control. JEV E and prM in supernatants of transduced cells were detected with mAb 4G2 or with the JEV antiserum, respectively. JEV VLPs were purified from supernatants of TRIP/JEV vector-transduced cells and analyzed by immunoblotting using anti-E mAb 4G2 and JEV antiserum. TRIP/GFP served as a negative control. The bands corresponding to prM, E or E^ΔTM^ and CNX are indicated with arrows to the right of the blots.

The antigenicity of recombinant JEV proteins was assessed by transducing HEK-293T cells with the TRIP/JEV.prME or TRIP/JEV.prME^ΔTM^ vectors (Fig 2A). An empty vector served as a control. At 48 h post-transduction, the intracellular form of the E protein was detected using the anti-E MAb 4G2 by IF assay. A similar staining pattern for E was observed in TRIP/JEV-transduced cells expressing prME or prME^ΔTM^. Immunoblot assays using mouse anti-JEV antisera detected recombinant prM and E in lysates from HEK-293T cells transduced with TRIP/JEV vectors (Fig 2B). We observed an efficient release of E protein to the supernatants of HEK-293T cells transduced with either TRIP/JEV vectors (Fig 2C, top). The presence of prM was only detected in supernatants from cells transduced with the TRIP/JEV.prME vector (Fig 2C, top). Because JEV prM and E have the capacity to self-assemble into VLPs, we assessed whether VLPs were secreted from HEK-293T cells transduced with TRIP/JEV vectors. The VLPs were detected by immunoblot assay using anti-E mAb 4G2 and anti-JEV immune serum (Fig 2C, bottom). Extracellular JEV VLPs containing prM and E accumulated in the supernatant of HEK-293T cells transduced with TRIP/JEV.prME vector but not with TRIP/JEV.prME^ΔTM^ vector. Thus, transduction of cells with TRIP/JEV.prME vector leads to efficient secretion of recombinant JEV VLPs.

### TRIP/JEV vectors induce anti-JEV antibody in mice

To evaluate humoral responses induced by the lentiviral TRIP/JEV vectors, adult BALB/c mice were inoculated with increasing doses of TRIP/JEV.prME or TRIP/JEV.prME^ΔTM^ (3 to 5 log_10_ TU per animal) by i.p. route. At 21 days post-immunization, sera were collected from each group of mice and pooled. Indirect ELISA was performed to detect anti-JEV IgGs using inactivated JEV particles as capture antigens (Table 1). NCA served as a control antigen. There was little to no antibody responses against JEV at TRIP/JEV vector doses lower than 5 log_10_ TU per animal. The dose of 5 log_10_ TU induced a significant production of anti-JEV specific antibodies with a mean titer reaching 1,600 for TRIP/JEV.prME and 400 for TRIP/JEV.prME^ΔTM^ (Table 1). At the highest dose (6 log_10_ TU) inoculated to mice, the mean titer of TRIP/JEV.prME antibody reached 10,000. The latter dose was not further used due to the too large volume of non-concentrated TRIP/JEV vector inoculated to mice by i.p. route. We therefore decided to select the unique dose of 5 log_10_TU in subsequent mouse immunizations. To determine the time course of anti-JEV production, BALB/c mice that received 5 log_10_TU of TRIP/JEV.prME or TRIP/JEV.prME^ΔTM^ were bled at 7, 14 and 21 days post-immunization (Table 1). Anti-JEV antibodies were detectable at day 14 of immunization and reached significant titers at day 21.

**Table 1:** A single dose of TRIP/JEV vector elicits anti-JEV antibody response.

	Immune sera	
**Viral dose** ^**1**^ **(TU)**	**TRIP/JEV.prME (21 days)**	**TRIP/JEV.prME** ^**ΔTM**^ **(21 days)**
10^3^	< 100	< 100
10^4^	100	100
10^5^	1,600	400
10^6^	10,000	n.d. ^3^
**Times post-immunization** ^**2**^ **(days)**	**TRIP/JEV.prME (10** ^**5**^ **TU)**	**TRIP/JEV.prME** ^**ΔTM**^ **(10** ^**5**^ **TU)**
7	< 100	< 100
14	400	200
21	1,600	400

^1^. Groups of 6-week-old BALB/c mice (*n* = 6) were inoculated with various doses of lentiviral vectors by the intraperitoneal route. Immune sera were collected at 21 days post-inoculation. The anti-JEV antibody titers in pooled sera were determined by indirect ELISA using inactivated JEV G3 as viral antigen. Typically, BALB/c mice inoculated with 3 log10 FFU of live JEV G3 virus have given anti-JEV antibody titers up to 64,000 at 21 days post-inoculation.

^2^. Groups of 6-week-old BALB/c mice (*n* = 6) were inoculated with 5 log_10_ TU of lentiviral vectors by the intraperitoneal route. Immune sera were collected at various days post-inoculation. The anti-JEV antibody titers in pooled sera were determined by indirect ELISA using inactivated JEV G3 as viral antigen.

^3^. n.d.: not done

To enhance the production of anti-JEV specific antibodies, groups of 12 mice previously immunized with 5 log_10_ TU of recombinant TRIP/JEV vectors received a booster dose of 5 log_10_ TU of homologous vectors bearing the VSV-G envelope protein of a different VSV strain (NJ), 4 weeks after the first inoculation. Immune sera were collected 3 weeks after the boosting inoculation and ELISA measurements on pooled sera showed a 40-fold increase in anti-JEV antibody titers reaching the mean titers of 64,000 for TRIP/JEV.prME and 16,000 for TRIP/JEV.prME^ΔTM^. It is of interest that the level of anti-JEV antibody in mice twice immunized with 5 log_10_ TU of recombinant lentiviral vector expressing JEV VLPs was similar to that obtained following a challenge with 3 log_10_ FFU of live JEV G3 RP-9.

### Reactivity of anti-TRIP/JEV antibodies against JEV prM and E

**Fig 3:**
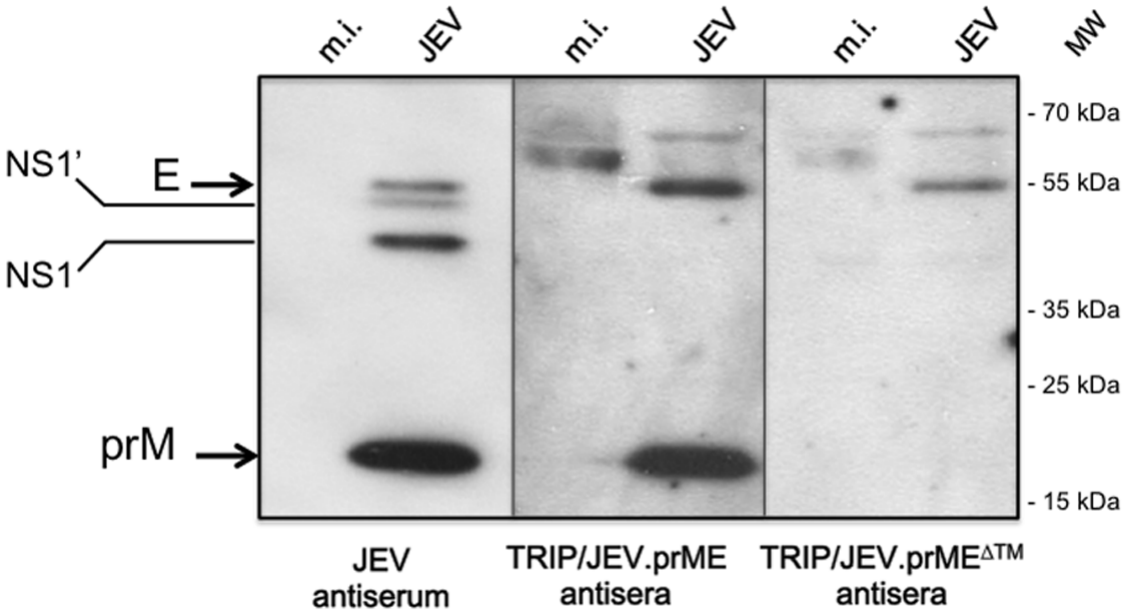
Reactivity of JEV prM and E with anti-TRIP/JEV antibody. Lysates obtained from SK-N-SH cells infected with JEV strain RP-9 (JEV) or mock-infected (m.i.) were tested with pooled immune sera (antisera) from BALB/c mice twice inoculated with 5 log_10_ TU of TRIP/JEV.prME or TRIP/JEV.prME vector by immunoblot assay. TRIP/JEV antisera were collected 3 weeks after the boosting inoculation. Mouse polyclonal serum directed against JEV strain RP-9 of G3 (JEV antiserum) served as a positive control. The bands corresponding to JEV E, NS1/NS1’ and prM proteins are indicated with arrows to the left of the blot.

**Fig 4:**
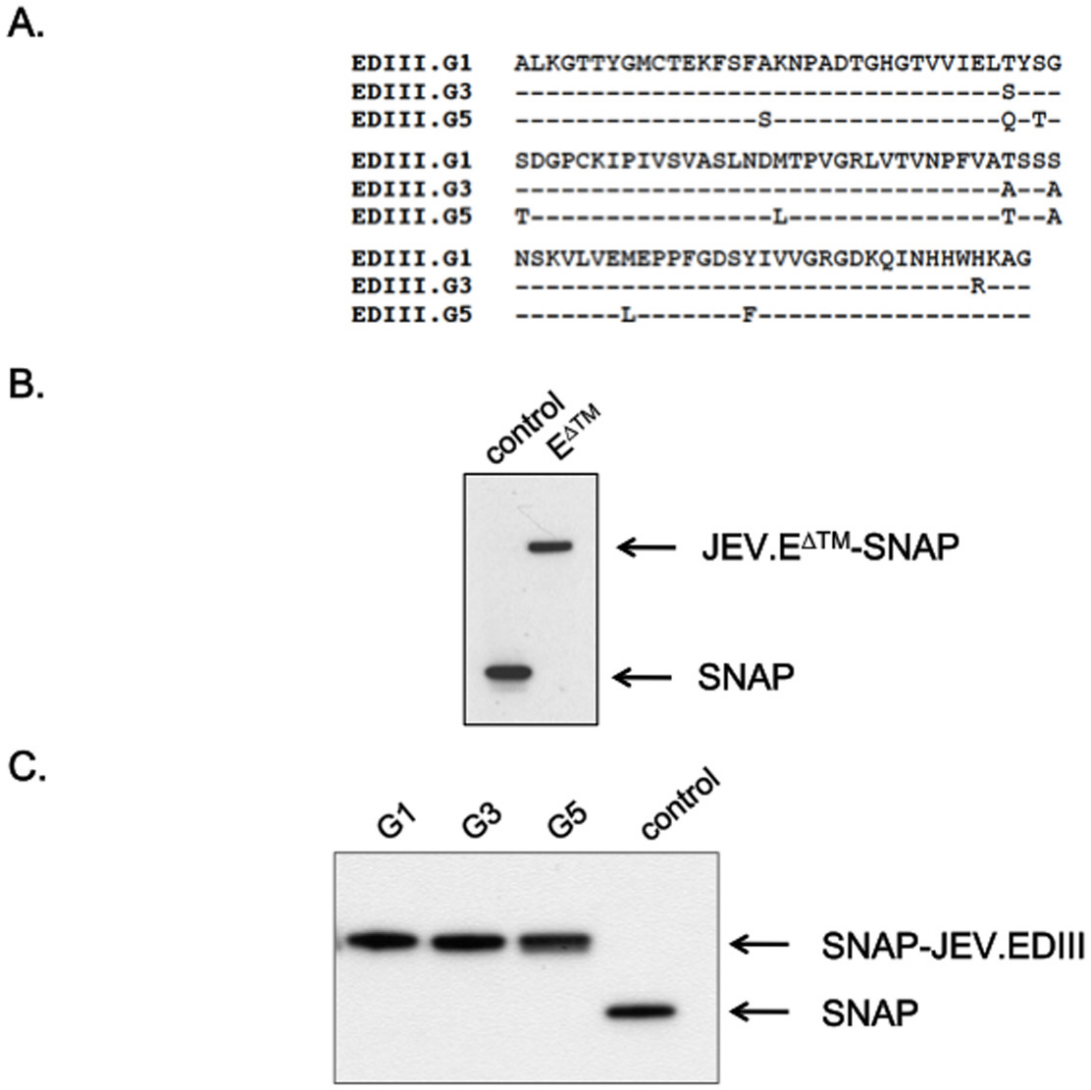
Analysis of recombinant JEV antigens. *In* (A), sequence alignment of EDIII from JEV of G1, G3, and G5. *In* (B, C), equal amount (100 ng) of highly purified recombinant proteins JEV.E^ΔTM^-SNAP (B) and SNAP-JEV.EDIII of G1, G3 or G5 (C) were analyzed by immunoblotting with an anti- SNAP tag antibody. Purified SNAP protein served as a control. The bands corresponding to JEV.E^ΔTM^-SNAP, SNAP-JEV.EDIII, and SNAP proteins are indicated with arrows to the right of the blot.

The reactivity of antibodies raised in mice after TRIP/JEV immunization was evaluated by immunoblotting using lysates of JEV-infected cells as viral antigens (Fig 3). Mice that received TRIP/JEV.prME displayed specific antibodies against prM and E whereas TRIP/JEV.prME^ΔTM^ antisera contained only anti-E antibody. Thus, the co-expression of prM and the soluble form of E failed to stimulate the production of anti-prM antibody in a mouse model. Indirect ELISA tests were performed to determine the anti-E antibody levels in sera from mice immunized with TRIP/JEV.prME or TRIP/JEV.prME^ΔTM^. The soluble form of JEV G3 E fused to SNAP tag (JEV.E^ΔTM^-SNAP) was used as a capture antigen of anti-JEV E IgGs. Given that flavivirus EDIII contains sub-type specific neutralizing epitopes, the recombinant SNAP-tagged EDIII proteins of G1, G3, and G5 were also used as capture viral antigens. Sequence alignment of the three EDIII identified a similarity of 96.3% between G3 and G1 and 92.5% between G3 and G5 (Fig 4A). Purity and specificity of recombinant JEV.E^ΔTM^-SNAP protein (Fig 4B) and SNAP-JEV.EDIII of G1, G3, and G5 protein (Fig 4C) were verified by immunoblotting using an antibody against SNAP-tag. Indirect ELISA showed that BALB/c mice that recovered from a lethal challenge with JEV G3 gave titers of anti-E antibody of 1,300 and anti-EDIII antibody from 1,000 to 4,000 (Table 2). BALB/c mice that received two doses of TRIP/JEV.prME^ΔTM^ or TRIP/JEV.prME elicited anti-E antibody titers with a similar range of about 1,000. Both lentiviral TRIP/JEV vectors were capable of inducing a similar level of anti-EDIII antibodies that are broadly reactive with different genotypes of JEV. It is of interest that immunization with TRIP/JEV vectors induced higher levels of anti-EDIII G5 antibody than live JEV G3.

**Table 2:** Reactivity of anti-JEV sera toward recombinant soluble E and EDIII proteins from JEV.

		Immune sera ^1^			
		Live JEV G3 ^2^	TRIP/JEV.prME ^3^	TRIP/JEV.prME^ΔTM^ ^3^
**JEV antigens**	**E** ^**ΔTM**^	1, 300	1,100	900
	**EDIII-G1**	4,000	8,000	8,000
	**EDIII-G3**	4,000	8,000	8,000
	**EDIII-G5**	1,000	4,000	4,000

^1^.The anti-JEV antibody titers in pooled immune sera were determined by indirect ELISA using recombinant JEV G3 E^ΔTM^ or recombinant EDIII from JEV of G1, G3, or G5 as viral antigens.

^2^. Immune sera were collected from groups of 3-week-old BALB/c mice (*n* = 6) at 21 days after intraperitoneal inoculation with 3 log_10_ FFU of JEV G3 (strain RP-9).

^3^. Immune sera were collected from groups of 6-week-old BALB/c mice (*n* = 6) at 21 days after intraperitoneal inoculation with 5 log_10_ TU of lentiviral vectors.

### *In vitro* cross-neutralizing activity of anti-TRIP/JEV antibody

A focus reduction neutralization test (FRNT50) was performed to evaluate the ability of TRIP/JEV vectors to elicit a neutralizing antibody response against JEV G3 (Table 3). Immune sera obtained from BALB/c mice that recovered from a lethal challenge with JEV strain RP-9 had a FRNT_50_ of 150. A weak FRNT_50_ titre of 10 was observed in mice inoculated with a single dose of 5 log_10_ TU of TRIP/JEV vector. A booster dose one month after the prime elicited JEV-neutralizing antibodies titers to 40 (TRIP/JEV.prME^ΔTM^) and 80 (TRIP/JEV.prME) (Table 3).

**Table 3:** Neutralizing activities of anti-TRIP/JE vector antisera.

		immune sera ^1^		
		Live JEV G3 ^2^	TRIP/JEV.prME ^3^	TRIP/JEV.prME^ΔTM^ ^3^
**JEV strain**	**G3**	150	80	40
	**G1/3**	140	180	140
	**G5/3**	50	60	30

^1^. The neutralization activities of pooled immune sera against three live JEV [G3 (strain RP-9), G1/3 chimera (strains CNS769_Laos_2009 and RP-9) and G5/3 chimera (strains XZ0934 and RP-9)] were evaluated using a standard FRNT assay in Vero cells. FRNT_50_ values representative of *n* = 3 independent experiments are given.

^2^. Immune sera were collected from groups of 3-week-old BALB/c mice (*n* = 6) at 21 days after intraperitoneal inoculation with 3 log_10_ FFU of JEV G3 (strain RP-9).

^3^. Immune sera were collected from groups of 6-week-old BALB/c mice (*n* = 6) at 21 days after intraperitoneal inoculation with 5 log_10_ TU of lentiviral TRIP/JE vectors.

**Fig 5:**
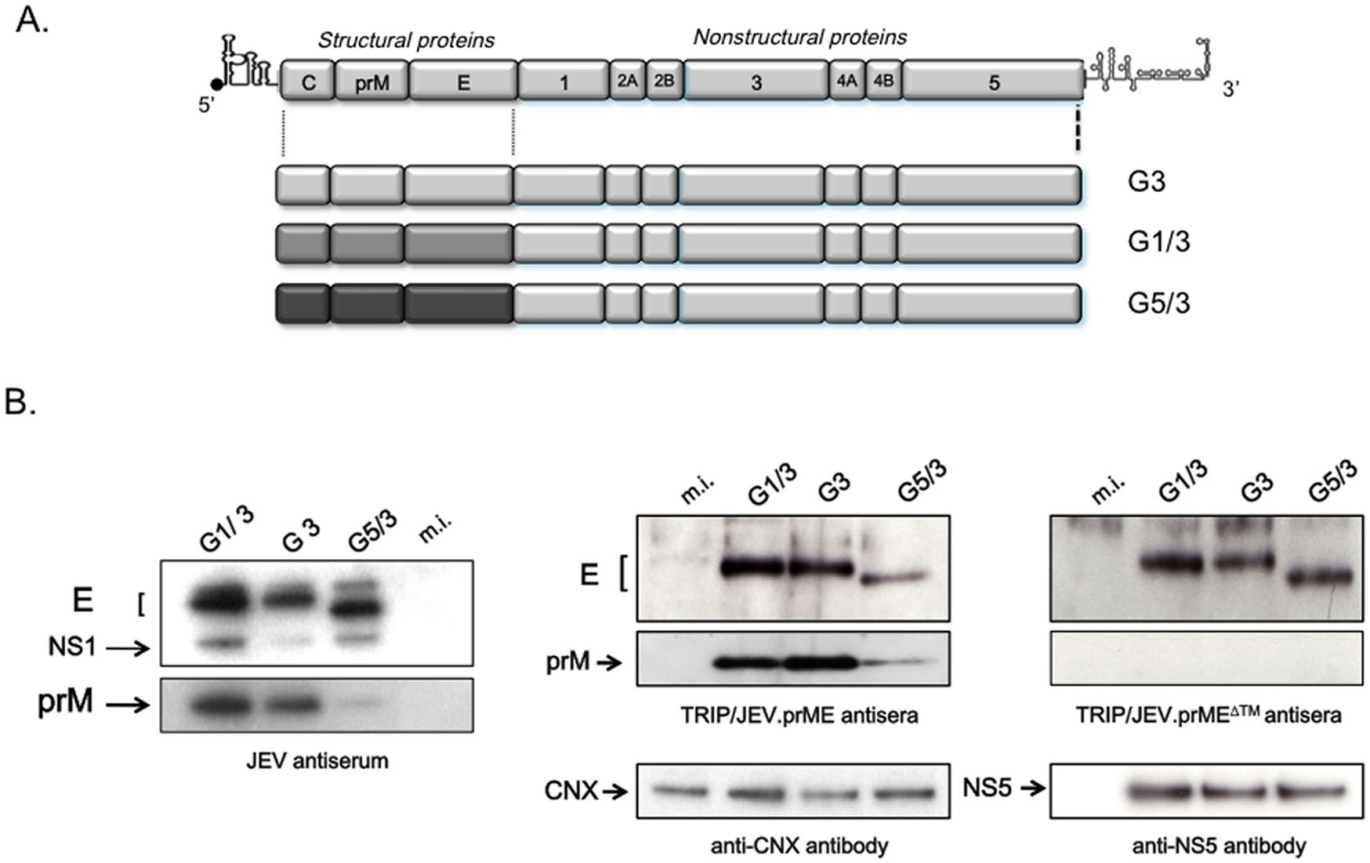
Construction strategy of chimeric live JEV and their recognition by TRIP/JEV antisera. *In* (A), schematic diagram of chimeric JEV G1/3 and G5/3 which include the sequences encoding C, prM and E from JEV of G1 or G5 into the backbone of JEV of G3. The parental JEV was designed as JEV G3. *In* (B), recognition of chimeric JEV proteins by JEV antiserum or TRIP/JEV antisera. Lysates obtained from SK-N-SH cells infected with parental JEV G3, chimeric JEV G1/3 and G5/3, or mock-infected (m.i.) were analyzed by immunoblotting with the indicated antibodies. Anti-JEV NS5 (NS5) and anti-calnexin (CNX) antibodies served as controls.

Recent studies addressed the ability of already existing G3 derived vaccines at protecting against strains belonging to distinct genotypes [32–34], and such testing should be systematically included when assessing newly designed JEV vaccines. We assessed the protective capacity of TRIP/JEV immunization against other circulating JEV genotypes, namely G1 and G5. To investigate this issue, we decided to substitute the region encoding C, prM and E into the infectious cDNA clone of JEV G3 by the counterpart from JEV G1 or G5 (Fig 5A). Since immunizations with the TRIP/JEV vectors are solely directed against JEV structural proteins, the contribution of non-structural proteins of JEV G1 and G5 was not explored. The growth of chimera JEV G1/3 or JEV G5/3 was comparable to that of parental JEV virus of G3 in cultured cell lines [29]. Immunoblot analysis showed that JEV G3 antisera recognized both prM and E from JEV G1 and G5 (Fig 5B, left panel). However, JEV G3 antisera weakly reacted with prM from the chimera JEV G5/G3. Essentially similar results were obtained when immunoblot assay was performed with anti-TRIP/JEV antisera (Fig 5B). The immune sera from BALB/c mice inoculated with TRIP/JEV.prME or TRIP/JEV.prME^ΔTM^ recognized the E protein from chimera JEV G1/G3 and G5/G3. Only TRIP/JEV.prME antisera reacted with prM from chimera JEV G1/G3 and a to lesser extent, JEV G5/G3. This lower reactivity was not due to a defect in JEV G5/3 protein accumulation since the reactivity of anti-JEV NS5 antibody was comparable amongst the different lysates of JEV-infected cells (Fig 5B, lower right panel). We observed that TRIP/JEV.prME^ΔTM^ was capable of inducing antibodies that can similarly react with the E protein from chimera JEV G1/3 and G5/3, thus suggesting that soluble E^ΔTM^ exhibits a greater propensity to generate anti-E antibodies that recognize conserved epitopes regardless of the genotype (Fig 5B).

FRNT assays were performed to evaluate the ability of TRIP/JEV vectors to elicit a neutralizing antibody response against JEV G1/3 or G5/3 (Table 3). Pooled immune sera obtained from BALB/c mice infected with JEV G3 gave had FRNT_50_ values of 140 and 50 against chimeric JEV G1/3 and G5/3, respectively. Immunized mice that received TRIP/JEV vectors developed neutralizing antibody that were also active against chimeric JEV G1/3 and G5/3 (Table 3). We noted that immunization with the TRIP/JEV.prME vector elicited slightly higher levels of neutralizing anti-JEV antibodies. The lower neutralization capability of TRIP/JEV-induced antibodies against chimera G5/3 correlated with the weaker reactivity of immune sera towards the prM and E proteins from JEV of G5 (Fig 5B). These data show that both TRIP/JEV vectors were capable of stimulating the production of anti-JEV antibodies that neutralize JEV G1 and G3, and to a lesser extent JEV G5.

### Passive transfer of TRIP/JEV antisera partially protects mice from JEV infection

**Fig 6:**
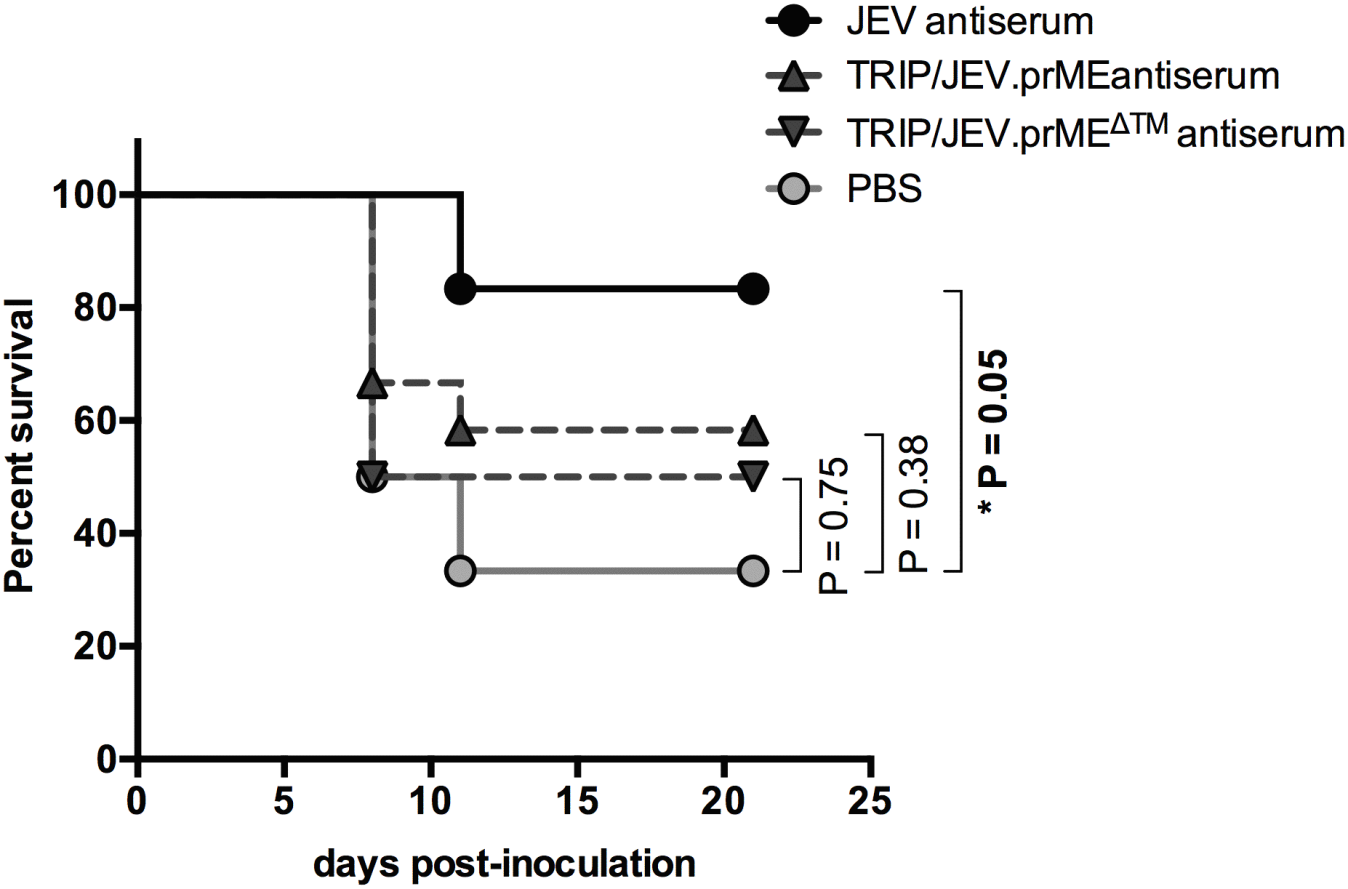
Protective capacity of TRIP/JEV antisera in a mouse model. Groups of 3-week-old C56BL/6 mice received i.p. inoculation with 0.1 ml of DPBS containing 0.01 ml of pooled immune sera collected from JEV-infected mice (JEV G3 antiserum) or TRIP/JEV-inoculated mice two months after boosting. Mice inoculated with DPBS (PBS) served as a group control. One day later, the mice were i.p. challenged with JEV strain RP-9 and observed daily for mortality. Survival was recorded for 21 days. The asterisk indicates that the differences between survival curves are statistically significant (*, *P* < 0.05, n.s.: *P* > 0.05).

We previously reported that inoculation of JEV G3 strain RP-9 to three-week-old C57BL/6 mice was lethal within one week [28]. Given that mouse susceptibility to JEV is age-dependent, we were unable to challenge animals following the long prime-boost vaccination period with TRIP/JEV vectors. Consequently, we decided to apply a protocol of passive transfer of TRIP/JEV antisera into three-week-old C57BL/6 mice. To address whether the humoral immunity elicited in mice after TRIP/JEV.prME or TRIP/JEV.prME^ΔTM^ vaccination was protective *in vivo*, groups of 3-week-old C57BL/6 mice received i.p. inoculation of 10 μl of pooled, heat-inactivated immune sera collected from TRIP/JEV-inoculated mice two months after boosting. Pooled immune sera of BALB/c mice inoculated with JEV G3 served as a positive control. A group of mice inoculated with PBS was included. One day after the passive transfer of antisera, the mice were i.p. challenged with a lethal dose of JEV G3. The animals were observed daily for clinical signs of illness and mortality over three weeks (Fig 6). Approximately 70% of the mice inoculated with PBS died within the 9–11 days post-challenge whereas administration of JEV immune sera induced a survival rate of 85% (*P* = 0.05). A survival rate of 50–60% was observed in mice after transfer of TRIP/JEV.prME or TRIP/JEV.prME^ΔTM^ antisera. While inoculation with a single dose of TRIP/JEV antisera did not confer satisfactory levels of protection in our mouse model, we do note that a slight level of protection was obtained.

### TRIP/JEV.prME induces the production of neutralizing anti-JEV antibodies in pigs

**Fig 7:**
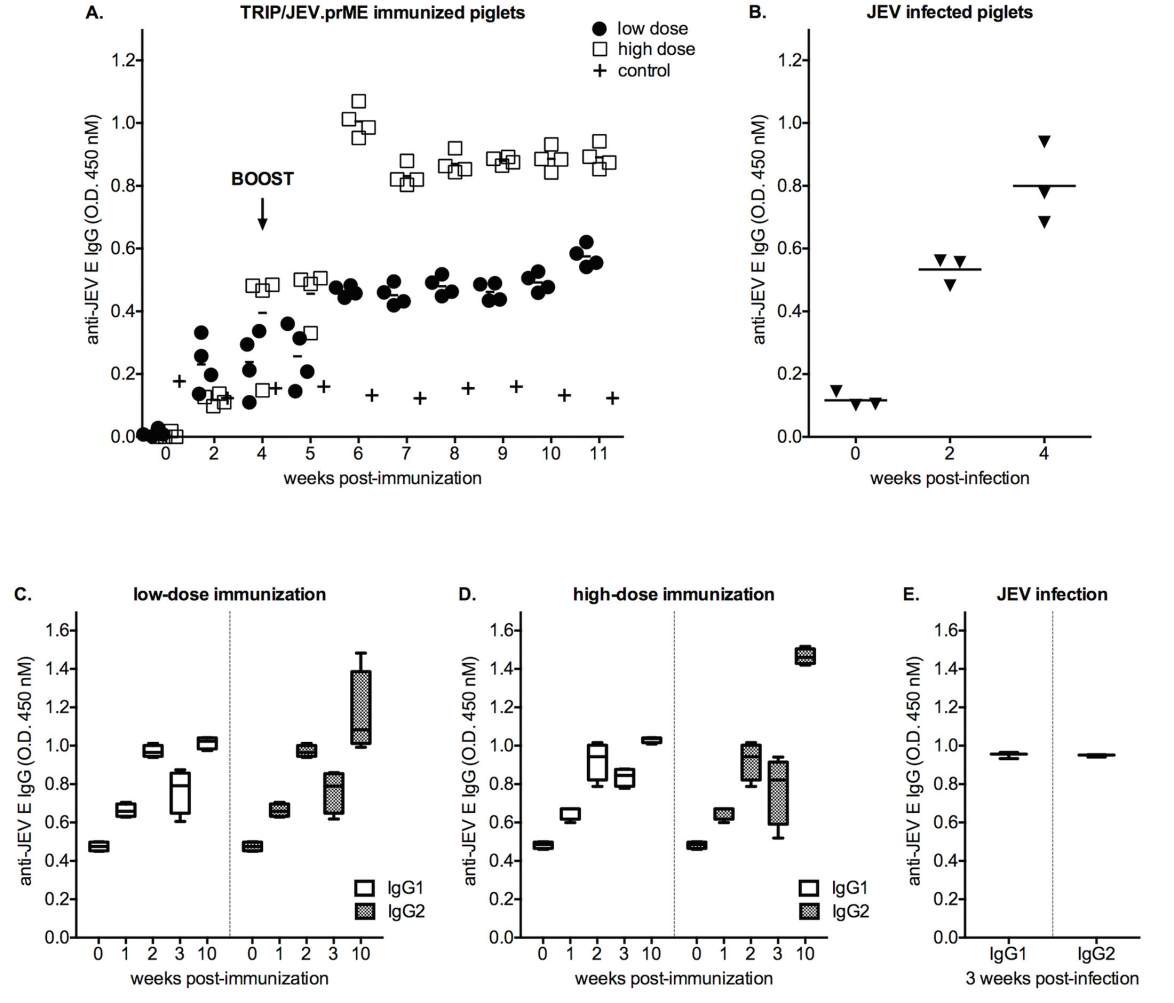
Anti-JEV IgG responses of piglets immunized with TRIP/JEV.prME. *In* (A), two groups of four piglets were immunized intramuscularly with 6 (low dose) or 7 log_10_ TU (high dose) of TRIP/JEV.prME. As a control, two animals were inoculated with either low or high dose of TRIP/GFP. Animals were boosted 4 weeks after primary immunization with the same initial dose (vertical arrow). Serum samples were collected weekly and tested at a dilution of 1:400 for the presence of anti-JEV E IgGs by indirect ELISA. *In* (B), a group of three animals were experimentally infected with JEV strain Nakayama. The immune sera were tested at a dilution of 1:400 for the presence of anti-JEV E IgGs by indirect ELISA. *In* (C, D), box plots of the anti-JEV E IgG1/IgG2 from 1 to 10 weeks after immunization with the low (C) or high (D) dose of TRIP/JEV.prME are depicted. The vertical arrow indicates the boost. *In* (E), the levels of anti-JEV E IgG1/IgG2 in immune sera from piglets infected with JEV strain Nakayama.

Because lentiviral based-expression of JEV VLPs is particularly efficient at triggering neutralizing antibody responses, we assessed the capacity of TRIP/JEV.prME to stimulate a protective humoral response in pigs. Groups of four 7-week-old piglets were immunized intramuscularly with 6 (low dose) or 7 (high dose) log_10_ TU of TRIP/JEV.prME (Fig 7). As a control, two animals received a low or high dose of a recombinant lentiviral vector expressing reporter GFP. Indirect ELISA using recombinant E^ΔTM^-SNAP protein as a viral antigen was used to assess the production of anti-JEV E antibodies in immunized pigs weekly (Fig 7A). The monitoring of the antibody responses during the first 4 weeks after the prime inoculation revealed an efficient production of anti-JEV E antibodies. Comparison of the low and high dose immunization did not show statistically significant differences in anti-JEV E antibody production over this time period. The levels of anti-JEV E antibodies was enhanced after the boost performed on week 4, and reached a plateau at least 1.5 month after the prime. When compared to the low dose, the high dose of TRIP/JEV.prME was more effective at eliciting a high level of specific antibody production (*P* = 0.028). As shown in the Fig 7B, the anti-JEV antibody titers induced 3 weeks after experimental infection of pigs with a single dose of live JEV were comparable to those stimulated in animals by a prime/boost immunization with 7 log_10_ TU of TRIP/JEV.prME lentiviral vector.

The isotyping of anti-JEV E antibodies showed that TRIP/JEV.prME stimulated the production of both IgG1 and IgG2 by 2 weeks after the prime, and was followed by a decline at week 3 even at the high dose (Fig 7C and 7D). The levels of both anti-JEV E IgG1 and IgG2 were similar to those observed in piglets challenged with JEV strain Nakayama at the week 3 of infection (Fig 7E). In animals primed with TRIP/JEV.prME, the boost at week 4 enhanced preferentially the production of IgG2 by 10 weeks after the prime regardless of the inoculated dose.

**Fig 8:**
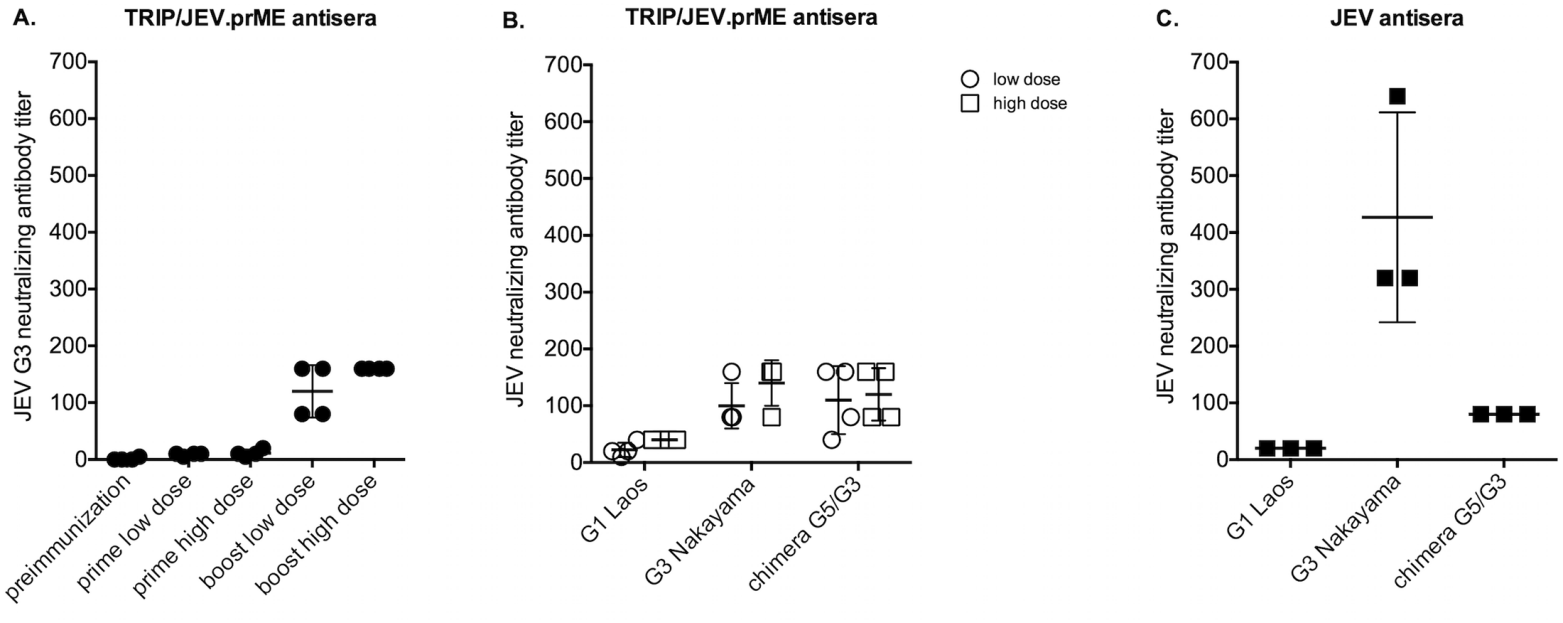
Neutralizing antibody response in piglets immunized with TRIP/JEV.prME. Sera from piglets immunized with a low or high dose of TRIP/JEV.prME were tested for neutralization ability against JEV by PRNT50. *In* (A), the piglet sera collected prior immunization, 3 weeks after priming or 6 weeks after the boost were tested against the JEV G3 strain RP-9. *In* (B), the TRIP/JEV.prME antisera collected after the boost were tested for their cross-neutralizing capacity against JEV G1 and G3 strains, and JEV chimera G5/G3. *In* (C) the neutralizing activity of anti-JEV antibodies from animals experimentally infected with JEV G3 strain Nakayama was tested against JEV G1, G3, and the JEV chimera G5/G3 by PRNT50.

The individual serum samples obtained from animals immunized with the lentiviral TRIP/JEV.prME vector were also examined for neutralizing antibodies at 3 weeks after the prime and at 6 weeks after the boost (Fig 8). Immunized piglets that received a single dose of 6 to 7 log_10_ TU of TRIP/JEV.prME developed neutralizing antibody titers ranging from 10 to 30 against the homologous JEV G3 strain RP-9 and reached titers up to 160 after the boost (Fig 8A). The higher dose of TRIP/JEV.prME induced a stronger anamnestic neutralizing antibody response.

Examination of the piglet immune sera revealed that, regardless of the inoculated dose, TRIP/JEV.prME elicited neutralizing antibodies against the Nakayama strain of JEV G3, the strain XZ0934 (tested using the JEV G5/G3 chimera) of JEV G5 and, to a lesser extent, the strain CNS769_Laos_2009 of JEV G1 (Fig 8B). Importantly, the pattern of neutralizing activity of anti-TRIP/JEV.prME antibody was similar to that observed in immune sera collected from a group of piglets experimentally infected with the JEV strain Nakayama (Fig 8C).

These results showed that TRIP/JEV.prME is able to elicit high titers of neutralizing antibodies in piglets that received two inoculations with 7 log_10_ TU of lentiviral vector with an interval of one month. Additionally, we found that TRIP/JEV.prME is capable of stimulating the production of anti-JEV antibodies that neutralize JEV G1 and G5.

## Discussion

Several vaccines against JEV are currently available to humans and for some animals such as horses and pigs: those are inactivated mouse brain-derived, inactivated cell culture-derived, live-attenuated and chimeric yellow fever virus-JEV vaccines [4, 7, 23, 32–44]. However, some of them lack induction of long-term immunity and live-attenuated vaccine strains carry a possible risk of reversion to virulence [4]. Also the cost effectiveness of JEV vaccines is considered as a major obstacle [7]. DNA vaccines were shown to be as successful as commercial live-attenuated vaccines at eliciting anti-JEV immune responses in a mouse [25] and non-human primate models [44]. While those vaccines were designed to express the prM and E proteins, the fact that they were DNA vaccines required greater production costs.

Lentiviral vectors represent a novel and attractive platform for gene-based immunization [20]. The ability of lentiviral vectors to efficiently transduce non-dividing dendritic cells allows a prolonged antigen presentation through the endogenous pathway, which in turns translates into the induction of strong, multi-epitopic and long lasting humoral as well as cellular immune responses. Consequently, an increasing number of pre-clinical studies show a great vaccine efficacy of lentiviral vectors in both infectious diseases and anti-tumor vaccination fields [19–21, 31, 45–47].

The purpose of our study was to evaluate the ability of two lentiviral TRIP-based vectors expressing the prM and E proteins from JEV, namely the TRIP/JEV.prME and TRIP/JEV.prME^ΔTM^ vectors, at eliciting protective humoral immune response in mice and piglets. We showed that JEV VLPs accumulated in the supernatant of cells transduced with TRIP/JEV.prME but not TRIP/JEV.prME^ΔTM^. Thus TRIP/JEV.prME expresses recombinant prM and E that are secreted efficiently, which represents a supplementary asset in the ability at eliciting anti-JEV antibody response. Mice inoculated with a single low dose (5 log_10_TU) of TRIP/JEV vectors developed JEV-specific IgGs and a booster dose one month after the prime resulted in a 40-fold increase in anti-JEV antibody titers. The reactivity of anti-JEV antibodies was documented by indirect ELISA and immunoblot assays. Mice immunized with TRIP/JEV.prME developed both anti-E and anti-prM antibodies whereas only anti-E antibodies were detected in sera of mice immunized with TRIP/JEV.prME^ΔTM^. The E protein acts as the main target for imparting protective immunity against JEV-related disease [23, 32], and its antigenic domain EDIII contains important epitopes that are recognized by neutralizing antibodies [48]. Further analysis of the recognition of JEV antigens by TRIP/JEV antisera showed that immunization with either TRIP/JEV.prME or TRIP/JEV.prME^ΔTM^ generated comparable levels of antibodies against the E protein, as well as type-specific epitopes located in its antigenic EDIII domain. Immunization with TRIP/JEV vectors induced neutralizing antibodies against JEV belonging to genotypes, G1, G3, and G5. Such observation is of particular importance at the period where it is evidenced that G1 JEV strains are replacing G3 strains [14], from which most vaccines were designed.

It is widely accepted that the humoral immune response is an essential component of protective immunity against JEV infection [23, 24]. Consistent with the notion that VLPs are suitable as vaccine against arboviral disease including Japanese encephalitis [5, 49], TRIP/JEV.prME was the more efficient lentiviral vector in the production of neutralizing anti-JEV antibodies that conferred partial protection after their passive transfer in mice challenged with JEV. Inoculation of two doses of 7 log_10_ TU with a one-month of interval of TRIP/JEV.prME vector in piglets was highly efficient at eliciting high titers of anti-JEV neutralizing antibody that are potentially able to protect pigs from JEV infection. TRIP/JEV.prME was capable of stimulating the production of anti-JEV antibodies that neutralize JEV G3 and G5, and, to a lesser extent, G1. The potential impact of JEV genotype change on vaccine potency has been estimated and immune sera obtained from pigs injected with a G3 vaccine showed lower strain-specific cross-neutralizing antibody titers against JEV of G1 [36]. Such observation led to the development of new veterinary vaccines for pigs specifically directed against this particular genotype of JEV [43]. Although in our hands the TRIP/JEV.prME vector elicited neutralizing antibodies against a G1 virus in pigs, we did note that their levels were lower when compared to the other JEV genotypes tested. However, neutralizing antibodies titers against JEV of G1 could reach 1:40, and thus could be sufficient to achieve protection in pigs. It could be nevertheless important to design alternate JEV antigen with a broader cross-reactivity against JEV strains of G1.

In this study, we demonstrated that immunization of pigs with a TRIP/JEV vector expressing JEV VLPs is particularly efficient at priming antigen-specific humoral immunity and triggers neutralizing antibody responses against the genotypes 1, 3, and 5 of JEV. The production of virus neutralizing antibodies is critical to protection against JEV infection in pigs [50] and a titer at least 1:10 is indicative of protective humoral immunity [51]. The titers of neutralizing antibodies elicited by the lentiviral TRIP/JEV.prME vector are sufficient to confer protection in domestic pigs against different genotypes of JEV and this could be of a great utility in endemic regions where more than one genotype circulates.
